# Assessment of Sodium and Potassium Intakes in Children Aged 6 to 18 Years by 24 h Urinary Excretion in City of Rabat, Morocco

**DOI:** 10.1155/2018/8687192

**Published:** 2018-08-01

**Authors:** Naima Saeid, Mohammed Elmzibri, Abdeslam Hamrani, Qandoussi Latifa, Hakim Belghiti, Hicham El Berri, Kaoutar Benjeddou, Amina Bouziani, Hasnae Benkirane, Youness Taboz, Asmae Elhamdouchi, Khalid El Kari, Hassan Aguenaou

**Affiliations:** ^1^Joint Research Unit in Nutrition and Food, URAC 39, (Ibn Tofaïl University-CNESTEN), Regional Designated Center of Nutrition (AFRA/IAEA), Rabat-Kénitra, Morocco; ^2^Military Hospital Mohammed V, Rabat, Morocco; ^3^Ministry of Health, Rabat, Morocco

## Abstract

**Background:**

The incidence of noncommunicable diseases (NCDs) has greatly increased, mainly due to high level of dietary sodium. Thus, reduction of sodium intake in population has been recognized as one of the most cost-effective strategies to reduce NCDs. The aim of this study was to estimate sodium and potassium consumption in a sample of Moroccan children as a baseline study to implement national strategy for salt intake reduction.

**Methods:**

The study was conducted on 131 children aged 6–18 years recruited from Rabat and its region. Sodium excretion and potassium excretion were measured on 24 h urinary collection, and the creatinine excretion was used to validate completeness of urine collections.

**Results:**

The average of urinary sodium was 2235.3 ± 823.2 mg/day, and 50% of children consume more than 2 g/d of sodium (equivalent to 5 g/day of salt), recommended by the WHO. However, daily urinary excretion of potassium was 1431 ± 636.5 mg/day, and 75% of children consume less than adequate intake. Sodium consumption increased significantly with age. Of particular interest, 46.7% of children aged 6–8 years and 49.3% of children aged 9–13 years consume more than the corresponding upper limits.

**Conclusions:**

Children have high sodium and low potassium status. There is evidence of the urgent need to implement a strategy for reduction of dietary sodium intake in Morocco.

## 1. Background

Worldwide, noncommunicable diseases (NCDs) are the leading cause of mortality and morbidity [1] accounting globally for 60% of all deaths and 43% of disease burden [2]. Evidence that diet is an important risk factor for NCDs has been reported by various researchers. Indeed, Western dietary patterns involving an over consumption of fatty, sugary, and salty foods proliferate in many emerging and developing countries [3].

Nowadays, it is widely accepted that an excessive intake of salt is associated with a broad range of NCDs, such as hypertension, cardiovascular diseases (CVDs), cancer, and osteoporosis [4, 5].

Most individuals suffering from higher blood pressure have been shown to have high sodium and low potassium consumptions [6]. In fact, several studies have shown a stronger relation between sodium/potassium ratio and blood pressure than sodium or potassium alone. Moreover, a high consumption of potassium, commonly from fruits and vegetables, can counteract the negative effects of high sodium intake on blood pressure [7, 8]. On the other hand, numerous studies have shown that blood pressure levels in infancy and childhood are moderately predictive for blood pressure later in life [9, 10].

Blood pressure in childhood or early adult life rises progressively through to middle age and predicts both the blood pressure level and presence of hypertension in later life [11]. So tracking of dietary habits from early childhood into adulthood has shown that children with extremely high levels of sodium and lower potassium intakes tend to maintain those levels over time [12, 13].

Therefore, diet in childhood can be a significant determinant of adult dietary habits even after several decades [14].

The World Health Organization (WHO) recommendation outlines the importance of prioritizing sodium intake reduction, as a main approach to reduce blood pressure and to decrease the risk of CVDs and strokes. Accordingly, the WHO recommends for adults the consumption of less than 2 g of sodium/day (5 g/day of salt) and more than 3.5 g of potassium/day [15]. For children, the reduction of sodium intake prevents and decreases risk of hypertension during childhood and adulthood. In this case, the maximum recommended level of sodium intake should be adjusted downward based on the energy requirements of children relative to those of adults [16]. Following these recommendations, the adequate intake for sodium for young people is set at 1.5 g/day (equivalent to 3.8 g of salt) without reducing the intake of other nutrients. The Institute of Medicine (IOM) sets the tolerable upper limit (UL) intake level at 2300 mg per day. The UL is the highest daily nutrient intake level that is likely to pose no risk of adverse health effects (e.g., for sodium, increased blood pressure). The UL depends on the age; for children aged from 6 to 8 years, the UL is set at 1900 mg/day, and for children aged from 9 to 13 years and 14 to 18 years, the ULs are set at 2200 mg/day and 2300 mg/day, respectively For instance, the IOM has reported that the adequate intake of potassium is 3.8 g/day for children aged 6–8 years and reaches 4.7 g/day for adolescents and adults [17].

Sodium intake can be estimated indirectly either from a questionnaire or food consumption data or directly by the measurement of urinary excretion. Because of the problems of underestimation of sodium intakes based on dietary surveys in most studies, 24-hour urinary sodium excretion has become the “gold standard” method of obtaining data on sodium and potassium intakes in population surveys [18]. However, the assessment of daily intakes by using 24 h dietary recall (three times) provides useful additional information on the nature of food consumed and helps to implement specific strategies based on studied population consumption.

However, fewer data are available on urinary sodium excretion in children and young people than that in adults, and these are mainly limited to the developed countries such as Europe and North America [19, 20].

This is mainly due to methodological difficulties in obtaining complete and valid urinary excretion for children [21]. The majority of studies on sodium and potassium consumption in children are based on food consumption, which is still difficult in this case.

In Morocco, high blood pressure is a public health problem; its prevalence was estimated at 33% according to the national survey carried out in 2000, and this prevalence is expected to continue to rise [22]. Thus, reduction of salt consumption would be a suitable strategy to fight against NCDs in Morocco. On the other hand, data on the daily intake of salt in Morocco through the diet of Moroccans remain limited. The most recent estimate was carried out by the Ministry of Health on 2008 before the addition of iodine to salt and revealed that the salt intake for adults reached 7–12 g/person/day [23].

Therefore, we have planned in this study to assess the consumption of sodium and potassium among a sample of Moroccan children as a part of a baseline survey (pilot study) to adapt and implement the WHO strategy to reduce the salt intake to the Moroccan context by using the 24 h urinary sodium/potassium excretion.

## 2. Materials and Methods

### 2.1. Subjects

This transversal study was conducted between September 2015 and June 2016. It was carried out among children aged 6–18 years, enrolling two primary schools and one middle school in Rabat and its nearest region.

The city of Rabat is the political and administrative capital of Morocco, and it is located on the Atlantic coast. The region of Rabat accounts about 1.6 million inhabitants, thus becoming the second largest agglomeration of the country after Casablanca [24]. The unemployment rate is 22.5% and the illiteracy is about 20.7% [25], whereas 16% of the population of Rabat are affected by poverty [26].

The study protocol was approved by the Ethics Committee for Biomedical Research, Faculty of Medicine and Pharmacy of Rabat, Morocco, and written informed consent was obtained from each parent of the recruited child.

### 2.2. Clinical Survey

A face-to-face interview was conducted with the participant's parents to collect information on health problems (exclusion criteria), date of birth, and consumption of medications and/or supplements.

### 2.3. Anthropometric Measurements

The anthropometric measurements were taken at schools according to the WHO recommendations [27]. Weight was measured using an electronic scale to the nearest 0.1/0.2 kg (Seca GmbH, Germany). Height was measured in the standing position, barefoot, using Shorr Board portable at the nearest of 0.1 cm (formerly Shorr Productions, LLC, USA). Waist circumference (WC) was measured at 0.1 cm threshold using an inelastic tape measure. The nutritional status was evaluated by different *Z*-scores (BMI *Z*-score, height-for-age *Z*-score, weight-for-age *Z*-score, and weight-for-height *Z*-score) calculated by the Anthroplus software [28].

The weight-for-age curve enables countries that routinely measure only weight to monitor growth throughout childhood. BMI-for-age is the recommended indicator for assessing thinness, overweight, and obesity in children aged 10–19 years.

### 2.4. Blood Pressure

Blood pressure was measured using an automatic vital signs monitor (OMRON M6 Comfort-HEM-7321 E). Measurements were completed in the sitting position after 10 minutes of rest with the child's arm positioned at the level of the heart on a rested table. The cuff was positioned on the child's arm by aligning the marked cuff artery indicator with the brachial artery. Three blood pressure readings were taken on the right arm at 1-minute intervals. The average of the three measurements will be used for analysis.

### 2.5. Urine Sampling

A single-timed 24-hour urine collection was obtained for estimation of electrolyte excretion. Instructions on urine collection were provided for parents, and simplified pictorial instructions were given to children. As requested, all participants were invited to discard the overnight urine to start the urine collection period with an empty bladder. During the following 24 hours, all urine voided was collected in 5-liter wide-mouth, rimmed polypropylene bottles. Start and finish urine collection times and any missed collections were also recorded on the 24-hour urine bottle. If the duration of the collection was not exactly 24 hours (but within 20–28 hours), urinary sodium, potassium, creatinine, and total volume were normalized to a 24-hour period [29].

Moreover, urine collections were being considered incomplete and then excluded if (1) the volume is less than 300 ml; (2) more than few drops of urine were lost during urine collection; (3) time outside of <20 hours or >28 hours; and (4) urinary creatinine was <280 mg/l or >2590 mg/l. In these cases, participants were excluded or invited to repeat the urine collection.

### 2.6. Urine Analysis

The total volume of urine collected was measured using a specially devised linear measuring scale, and an aliquot (20 ml) from the complete samples was stored at −20°C until analysis. Urinary sodium and potassium concentrations were assessed using inductively coupled plasma mass spectrometry (ICP-MS; Thermo Scientific X-SERIES 2); coefficient of variation was 1.5% for sodium and 2.5% for potassium. An international reference material (Seronorm TM Trace Elements Urine) was used to control and validate the results. Total sodium and potassium excretions during 24 hours are calculated by multiplying the obtained concentrations by the volume of the collected urine and reported to 24-hour urine excretion [30]. Daily sodium and potassium intakes were expressed in grams, and sodium concentrations were converted to the salt equivalent (g) using the conversion factor 2.54 [21]. Na^+^/K^+^ ratio was also calculated.

Urinary creatinine excretion was analyzed by the kinetic approach according to Jaffé method [31], using the Cobas C311 (Roche Diagnostic, Meylan, France).

### 2.7. Statistical Analysis

All statistical analyses were carried out using SPSS for Windows, version 20 (SPSS Inc, Chicago). Variables were expressed as mean ± standard deviation (SD) or as interquartile range. The normality of data was checked, and the association between sodium intake and the studied variables was assessed by the Kolmogorov–Smirnov test. The significance level was established as 5% (<0.05). Independent samples *t*-test was used to compare boys with girls within each age group for continuous data. The percentages of children above the maximum salt intake recommendations are reported.

The distributions of children by age groups were made according to the Institute of Medicine (IOM) children stratification [17].

## 3. Results

### 3.1. Participants

**Figure 1:**
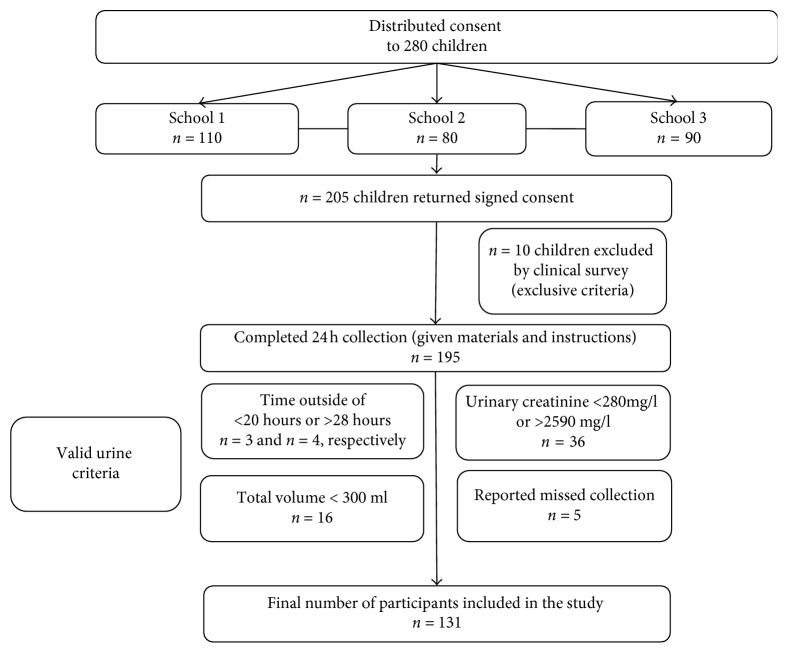
Flowchart of the study.

The flow diagram of the study is reported in Figure 1. A total of 205 children were recruited from the 3 schools. Among them, 10 were excluded for (1) nonattendance on days when 24 h urinary collection was collected; (2) having a disease that might affect the results (diabetes, renal disease, etc.); (3) being under special diet; and (4) taking antidiuretic drugs the week previous to the study. Complete information regarding clinical survey, anthropometric data, and 24 h urine samples was obtained from 195 eligible children. Among them, 64 children were withdrawn for nonvalid urine sampling: 5 children have reported that the collection time was less than 20 hours or more than 28 hours; 36 children have abnormal creatinine values; 6 children have collected less than 300 ml of urine; and 5 children have reported that they missed more than 2 times of collection. Therefore, only 131 participants have been retained in the study.

### 3.2. Characteristics of Study Participants

In our study, boys represent 51.9% (68/131) and girls 48.1% (63/131), with a sex ratio of 1.08. The mean age was 9.8 ± 2.4 years. Among the 131 children, 19.1% aged 6–8 years (25/131), 68.7% aged 9–13 years (90/131), and 12.2% aged 14–18 years (16/131) (Table 1).

The characteristics of growth parameters for the studied children are presented in Table 1. For overall recruited children, the mean weight was 32.7 ± 11.3 kg and the average height was 137 ± 14.4 cm. For nutritional status, the BMI-*Z*-score indicates that 9.9% children are underweight, 12.2% present overweight status, and 8.4% are obese. Statistical analysis showed no significant difference between boys and girls for all parameters.

### 3.3. Assessment of Sodium and Potassium Status

Excretion of urinary sodium and potassium is summarized in Table 2. The average of urine creatinine excretion was 852.8 ± 352.7 mg/day. The mean of daily urinary excretion of sodium in the whole group was 2235.3 ± 823.2 mg/day (equivalent to 5667.9 ± 2077.7 mg/day of salt). Overall, 50% of children consume more than 2 g/day of sodium (equivalent to 5 g/day of salt). However, daily urinary excretion of potassium was 1431 ± 636.5 mg/day, and 75% of children consume less than adequate intake. The mean sodium-to-potassium ratio was 1.7 ± 0.7, so children consume double the amount of the adequate intake. A comparison of daily sodium and potassium urinary excretion, sodium/potassium ratio, and creatinine level between boys and girls are also reported in Table 2 and showed no significant statistical difference.

Table 3 provides the distribution of sodium intake according to age, gender, and nutritional status. Data clearly showed that for all age groups, sodium intake exceeds the upper limits (ULs) according to ages' energetic needs. Sodium intake increases with age, children aged 6–8 years consume an average of 1800.0 (1450.1, 2145.3) mg/day, and 46.7% of children consume over the UL. Children aged 9–13 years consume 2193.4 (1843.6, 2793.8) mg/day, and more than 49.3% have over UL. Finally, 26.7% of children aged 14–18 years have over ULs and consume an average of 2138.0 (1876.7, 2392.5) mg/day. Differences between age groups are statistically significant (*p* < 0.001). Distribution of sodium intake and the corresponding proportion of children exhibiting sodium status over the UL level, according to gender and nutritional status, showed no statistically significant difference (*p* < 0.05).

## 4. Discussion

Worldwide, there are limited data on sodium and potassium intakes in children and young people and are limited to high-income countries such as Europe and North America [32]. In Morocco, and to our best knowledge, this study is the first investigation to assess sodium and potassium status in children.

In this study, the assessment of sodium status in children aged 6–18 years old, with 24-hour urinary sodium excretion, clearly showed that the average daily sodium consumption in Morocco exceeds the recommended values for both boys and girls. With 24-hour urinary sodium excretion, the mean sodium concentration was 2235.3 ± 823.2 mg/day (equivalent to 5667.9 ± 2077.7 mg/day of salt).

Our results are in agreement with previously reported data worldwide using the 24 h urinary excretion approach, indicating a highly average consumption of sodium, particularly in industrialized countries. In Europe, the mean of sodium intake among children was included between 2400 mg/day and 3000 mg/day [33–36]. In USA and China, the sodium intake is much higher, and reported data showed the mean sodium intake was 3100 mg/day and 3400 mg/day, respectively [37, 38]. Up to now, the highest mean of dietary sodium intake was reported in Chinese children aged 12–16 years from rural Shanxi, with a mean intake of 4 g/day [37].

In 2004, the Canadian Community Health Survey (CCHS) has evaluated sodium intake in a population of children aged 9–18 years and have clearly showed that sodium intakes are too high and exceed the ULs for 99% of boys and 91% of girls, increasing the risk to develop adverse health effects immediately or at adult age [36].

On the other hand, it is widely accepted that potassium has a critical role in many physiological processes, and potassium needs are widely discussed. In 2010, the American Dietary Guidelines identified potassium as a nutrient to be increased in the diet [39]. For instance, the Institute of Medicine (IOM) has reported that the adequate intake of potassium is 3800 mg/day for children aged 6–8 years and reaches 4700 mg/day for adolescents and adults [17].

In our study, potassium consumption is low for almost children and does not reach the recommended intake levels for all age groups. Overall, the average urinary potassium concentration was 1431 ± 636.5 mg/day, and more than 97% of children consume less than adequate intake. The average sodium-to-potassium ratio is 1.7 ± 0.7, so sodium is overconsumed compared to low potassium consumption.

Recently, Campanozzi et al. have reported similar results in Italy with an estimated daily potassium intake of 1530 mg/day for boys and 1400 mg/day for girls, much lower than the age-specific adequate intakes. Moreover, over 96% of the boys and 98% of the girls have a potassium intake lower than the recommended adequate intake [33]. Equivalent situation was reported in the USA. Indeed, the average of dietary potassium intake of the U.S. children aged 6–11 years and 12–19 years was 2225 mg/day and 2100 mg/day, respectively [40].

This study highlights that our recruited children have high level of sodium and low level of potassium intakes, as compared to recommended daily intakes. During last decades, Morocco has registered a nutritional transition, leading to changes in dietary habits and lifestyle modifications, especially at the biggest cities such as the Rabat region. Accordingly, the processed food and restaurant/fast food have been dramatically increased. In Morocco, food was usually prepared at home, but growing interest is currently given to a commercially processed food, especially by young people, under 25 years, representing 55% of the whole population [41].

A considerable part of the salt consumed in Morocco comes from the important bread consumption by adults. Indeed, most bakeries use between 15 and 20 g of salt/kg in bread preparation, and in Moroccan diet, bread is excessively consumed during meals, with an average of 500 g/day/person, which leads to a daily intake of 8 to 9 g of salt through bread alone [42]. For children, salt coming from bread would be slightly lower due to less consumption of bread. Worldwide, dietary sodium comes mainly from pre-prepared meals, snacks, and fast food. According to the UK National Food Survey data collected in 2000, cereal products (including bread, other baked goods, and breakfast cereals) accounted for the greatest proportion (38%) of household sodium intake, whereas 10% are added at home, during cooking or at table, and only 10% are naturally brought by food [43]. In the UK, children and adolescents, aged 4–18 years, surveyed for the UK Diet and Nutrition Investigation in 1992, showed that the major sources of sodium reflect those for adults (cereals contribute 38–40% and meat products contribute 20–24%) [44].

Fruits and vegetables are considered as the main sources of potassium. In Morocco, consumption of fruits and vegetables is very low and is more pronounced in children, consuming less than 374 g/person/day, giving rise to the lower potassium intake in Moroccan children [45].

This inadequate status of sodium and potassium in Morocco reflects the worldwide situation with an excess of sodium and deficiency of potassium intakes. According to this worrying situation, the WHO has developed and is implementing a global strategy and effective policies for salt consumption reduction, targeting the main sources of dietary sodium for all age groups. In this strategy, modification of dietary habits is also pointed out through nutritional education of the whole population, especially children, including enhancing legume and fruit consumption, limiting fast food and processed products, avoiding the use of table salt, and so on [46].

Excessive sodium and deficient potassium intakes could lead to metabolic dysfunctions in children. Tracking of dietary habits from early childhood to adulthood has shown that children with extremely high levels of sodium intake tend to maintain those levels over time [12]. Thus, close monitoring of these children is needed for better management of their health.

Moreover, nutritional campaigns, aiming to increase the awareness about correct sodium and potassium intakes, should focus on children and adolescents as a major target in the framework of the population strategies for prevention of noncommunicable diseases.

The present study is very informative and has much strength mainly related to the technical approach used for assessment of sodium and potassium. In fact, evaluation of sodium and potassium was done by an objective indicator, 24 h urinary excretion, using a validated protocol for 24 h urine collection. However, the main limitation of the study is the assessment of sodium and potassium status in only one 24 h urine excretion per child. Moreover, the study was done only on children from Rabat and its region, and further studies on representative children from the whole country are necessary to draw consistent conclusions regarding the status of sodium and potassium in Moroccan children and to elaborate more adapted strategies to fight against associated NCDs.

## 5. Conclusion

In conclusion, the present study gives evidence that children from Rabat and its nearest region have inadequate sodium/potassium status, with higher sodium and lower potassium intakes compared to recommended values. These results give an idea about the sodium and potassium status in Moroccan children and strongly support the urgent need to implement a systematic strategy for the reduction of dietary sodium intake in Morocco. This study must be completed by the assessment of sodium and potassium intakes in Moroccan adults to have a global picture on these micronutrients' status and a valid baseline to follow up the population and evaluate the WHO strategy for reduction of salt consumption in Morocco.

## Figures and Tables

**Table 1:** General characteristics of the studied population.

	Total (*n*=131)		Boys (*n*=68)		Girls (*n*=63)		*p* values
	Mean ± SD	Median (interquartile range)	Mean ± SD	Median (interquartile range)	Mean ± SD	Median (interquartile range)	
Age (years)	9.8 ± 2.4	9.7 (8.4, 11.2)	9.8 ± 2.4	9.4 (8.1, 11.0)	10.5 ± 2.6	9.9 (8.9, 11.4)	0.1
Weight (kg)	32.7 ± 11.3	30.4 (25.0, 38.4)	32.7 ± 11.3	29.5 (24.8, 39.4)	34.1 ± 12.4	31.6 (25.9, 37.9)	0.4
Height (cm)	137 ± 14.4	137.0 (130, 144.9)	137 ± 14.4	137.2 (126.7, 144.0)	138.5 ± 13.8	136 (130, 148)	0.5
BMI	17.1 ± 3.6	16.5 (14.6, 18.6)	17.1 ± 3.8	16.08 (14.5, 18.2)	17.3 ± 3.5	16.8 (14.8, 18.8)	0.7
BAZ	−0.1 ± 1.6	0.05 (−1.1, 0.7)	−0.1 ± 1.8	0.05 (−1.1, 0.75)	−0.15 ± 1.3	0.03 (−1.9, 0.9)	0.3
Thinness	9.9	—	10.3	—	9.5	—	
Normal	69.5	—	72.1	—	66.7	—	
Overweight	12.2	—	8.8	—	15.9	—	0.8
Obesity	8.4	—	8.8	—	7.9	—	
Blood pressure							
Systolic (mmHg)	98.5 ± 11.7	98 (90–107)	98.1 ± 12	98.3 (88.4–108.4)	98.9 ± 10.1	99 (91.3–107)	0.7
Diastolic (mmHg)	69.3 ± 9.9	70 (61–77.5)	68.8 ± 10.7	70 (60–77.8)	69.8 ± 9.01	70 (62–77.3)	0.5

*p* values by one-way ANOVA for means or Mann–Whitney *U* test for medians. Results are presented as mean ± standard deviation or proportion (%); BMI (body mass index), BAZ (BMI *Z*-score of body mass index for age), and *Z*-scores were determined according to [27].

**Table 2:** Urinary sodium and potassium excretion according to the sex.

	Total (*n*=131)		Boys (*n*=68)		Girls (*n*=63)		*p* value
	Mean ± SD	Median (interquartile range)	Mean ± SD	Median (interquartile range)	Mean ± SD	Median (interquartile range)	
Sodium (mg/d)^*∗*^	2235.3 ± 823.2	2094.2 (1640.3, 2498.0)	2184.3 ± 783.3	2057.0 (1600.0, 2462.5)	2290.3 ± 867.2	2138.0 (1765.3, 2669.1)	0.4
Potassium (mg/d)	1431 ± 636.5	1251.6 (994.3, 1813.0)	1469.4 ± 721.3	1217.7 (902.9, 1940.6)	1389.5 ± 532.8	1288.3(1022.6, 1288.3)	0.47
Sodium-to-potassium ratio	1.7 ± 0.7	1.7 (1.2, 2.1)	1.7 ± 0.7	1.6 (1.2, 2.03)	1.8 ± 0.7	1.7 (1.2, 2.2)	0.4
Salt (mg/d)	5667.9 ± 2077.7	5316.3 (4166.4, 6345.0)	5548.3 ± 1989.6	5224.7 (4063.9, 6254.7)	5797.0 ± 2177.5	5430.6 (4483.8, 6779.4)	0.5
Creatinine (mg/d)^*∗*^	852.8 ± 352.7	842 (620, 1020)	853.4 ± 324.4	866 (609.5, 1010.3)	852.1 ± 329.7	789 (621, 1058)	0.9
Volume (ml/d)^*∗*^	0.8 ± 0.4	0.8 (0.7, 1.1)	0.9 ± 0.4	0.8 (0.6, 1.01)	0.9 ± 0.3	0.8 (0.7, 1.0)	0.3

^*∗*^Variables are not normally distributed (Kolmogorov–Smirnov test). *p* values by one-way ANOVA for means or Mann–Whitney *U* test for medians.

**Table 3:** Distribution of sodium excretion according to age groups, sex, and nutritional status.

	Sample, *n* (%)	Sodium intake (mg/day) Median (interquartile range)	UL (mg/d)	Proportion over UL level, *n* (%)	*p* value
Age group					
6–8 y	45 (22.8)	1800.0 (1450.1, 2145.3)	1900	21 (46.7)	<0.001
9–13 y	71 (63.6)	2193.4 (1843.6, 2793.8)	2200	35 (49.3)	
14–18 y	15 (13.6)	2138.0 (1876.7, 2392.5)	2300	4 (26.7)	
Gender					
Boys	68 (41.2)	2057.0 (1600.0, 2462.5)	2300	40 (58.8)	0.487
Girls	63 (58.8)	2138.0 (1765.3, 2669.1)	2300	35 (55.6)	
Anthropometric status					
Thinness	13 (9.9)	1988.5 (1545.8, 2851.9)	2300	4 (26.5)	0.678
Normal	91 (69.5)	2107.4 (1733.9, 2478.2)	2300	48 (52.5)	
Overweight	16 (12.2)	2044.3 (1787.5, 3153.6)	2300	10 (62.5)	
Obese	11 (8.4)	2076.3 (1624.0, 2373.1)	2300	3 (31.5)	

UL: the upper limit refers to the highest daily level of sodium that is likely to pose no risk of adverse health effects to almost all individuals in the general population. The UL is not a recommended intake, and there is no apparent benefit to consuming levels of sodium above the adequate intake (AI). Source: Institute of Medicine [17]. *p* values are determined using the Kruskal–Wallis test for medians.

## Data Availability

The data used to support the findings of this study are available from the corresponding author upon request.
